# Protective effects of astaxanthin against ischemia/reperfusion induced renal injury in mice

**DOI:** 10.1186/s12967-015-0388-1

**Published:** 2015-01-27

**Authors:** Xuefeng Qiu, Kai Fu, Xiaozhi Zhao, Yanting Zhang, Yimin Yuan, Shiwei Zhang, Xiaoping Gu, Hongqian Guo

**Affiliations:** Department of Urology, Affiliated Drum Tower Hospital, School of Medicine, Nanjing University, Nanjing, 210008 China; Institute of Urology, Nanjing University, Nanjing, 210093 China; State Key Laboratory of Pharmaceutical Biotechnology, Nanjing University, Nanjing, 210093 China; Department of Anesthesiology, Affiliated Drum Tower Hospital, School of Medicine, Nanjing University, Nanjing, 210008 China

**Keywords:** Renal transplantation, Kidney, Ischemia/reperfusion, Oxidative stress, Astaxanthin, Antioxidant

## Abstract

Astaxanthin (ATX) is a powerful antioxidant that occurs naturally in a wide variety of living organisms. Previous studies have shown that ATX has effects of eliminating oxygen free radicals and can protect organs from ischemia/reperfusion (IR) induced injury. The present study was designed to further investigate the protective effects of ATX on oxidative stress induced toxicity in tubular epithelial cells and on IR induced renal injury in mice. ATX, at a concentration of 250 nM, attenuated 100 μM H_2_O_2_-inudced viability decrease of tubular epithelial cells. In vivo, ATX preserved renal function 12 h or 24 h post IR. Pretreatment of ATX via oral gavage for 14 consecutive days prior to IR dramatically prevented IR induced histological damage 24 h post IR. Histological results showed that the pathohistological score, number of apoptotic cells, and the expression of α-smooth muscle actin were significantly decreased by pretreatment of ATX. In addition, oxidative stress and inflammation in kidney samples were significantly reduced by ATX 24 h post IR. Taken together, the current study suggests that pretreatment of ATX is effective in preserving renal function and histology via antioxidant activity.

**Electronic supplementary material:**

The online version of this article (doi:10.1186/s12967-015-0388-1) contains supplementary material, which is available to authorized users.

## Introduction

Kidney transplantation presents one of the gold standard therapies for end-stage renal diseases. Given the storage of organs from decreased donors available for transplantation, transplants using so-called “marginal” organs have increased despite their reduced viability and increased immunogenicity compared with living donor renal allografts [1,2]. Ischemia/reperfusion (IR), an inevitable consequence of kidney transplantation, is one of the major contributors to the development of primary injury, and delayed graft function in kidney transplantation, leading to worse long-term function [3,4].

Although the mechanisms by which organ damage occurs in IR-induced renal injury are incompletely understood, it has been suggested that oxidative injury following IR most likely contributed to the pathogenesis of IR [5]. During the ischemia phase, ischemia-induced hypoxia could induce ATP depletion and hypoxanthine accumulation. After reintroduction of blood flow, reactive oxygen species (ROS) are generated [6], leading to tubular apoptosis/necrosis and local inflammation [7]. As a result of oxidative and inflammatory injury caused by IR, tubular epithelial cells might undergo profound functional alterations including losing tight junctions and epithelial-to-mesenchymal transition (EMT) [8,9]. Previous reports have demonstrated that deficiency of antioxidant could exacerbate IR injury and antioxidants such as superoxide dismutase, catalase, or vitamin E had beneficial effects for IR injury [10,11], supporting oxidative stress as a major contributor in the development of IR-induced injury.

Astaxanthin (ATX) is a carotenoid pigment naturally existing in seafood, such as salmon, shells of crabs and shrimps, as well as a wide variety of plants and algae [12]. The United States FDA approved ATX as a feed additive for the aquaculture industry in 1987 and approved its use as a dietary supplement in 1999 [13]. The molecular structure of ATX is similar to that of β-carotene (Additional file 1: Figure S1), which gives ATX antioxidant capacity [14]. ATX exhibits free radical scavenging activity and protect against lipid peroxidation and oxidative damage to cell membranes, cells, and tissues, suggesting ATX as a powerful biological antioxidant [15]. Unlike carotenoids, ATX has oxygenated groups on each ring structure (Additional file 1: Figure S1), making ATX highly polar and dramatically enhancing its membrane function to protect against degenerative conditions. This makes ATX a significantly greater antioxidant than β-carotene [16]. Previous studies on animal models have shown protective effects of ATX in IR-induced liver [17], brain [18,19], or cardiovascular [20] injury by reducing oxidant-induced damage. However, the protective effect of ATX against IR-induced renal injury still remains unknown.

The present study was therefore designed to examine the therapeutic effect of ATX in IR-induced renal injury using an IR model in mice. We hypothesize that pretreatment of ATX could offer protective effects against IR-induced renal injury by reducing ROS, tubular apoptosis, inflammation, and EMT. To the best of our knowledge, this is the first study designed to explore the feasibility of using ATX, a powerful antioxidant, for IR-induced renal injury.

## Materials and methods

### Cells culture and treatment

Human tubular epithelial cells (HTECs) were obtained from Dr. Hao Chen (National Clinical Research Center of Kidney Diseases, Nanjing University, Nanjing, China) and cultured in Roswell Park Memorial Institute 1604 medium (RPMI 1640, Life Science, Shanghai, China) supplemented with 10% fetal bovine serum (FBS, Life Science). Culture incubator was set at 37°C with 5% CO_2_. In order to produce oxidative stress, H_2_O_2_ was freshly prepared prior to each experiment. Free type of ATX (≥97%, Sigma-Aldrich, St. Louis, MO, USA) was dissolved in 0.5% DMSO. The cells were pretreated with ATX at concentration of 250 nM for 24 h followed by exposure to 100 μM of H_2_O_2_ in the presence of the same concentrations of ATX for another 2 h.

### Cell viability assay

Cell viability was evaluated by the Cell Counting Kit-8 (CCK-8, Life Science) test. Five thousand cells were seeded into a 96-well culture plate and cultured in the serum-free growth medium. According to the instructions provided by the manufacturer, 100 μl of CCK-8 solution were added to each well and the cells were incubated for a further 2 h. After indicated time points, absorbance was measured at 450 nm with a multidetection micro-plate reader (Versamax, Downingtown, PA, USA).

### Animals and treatment

All experimental protocols conducted on animals were performed in accordance with the standards established by the Institution Animal Care and Use Committee at Nanjing University. Male ICR mice weighing 20-25 g were housed in stainless steel cages and given free access to food and water. Mice were randomly divided into four groups: The sham group received olive oil via oral gavage for 14 consecutive days [17] before the sham surgery (n = 6); the sham + ATX group received 5 mg/kg/day ATX (3,3-dihydroxy beta, β-carotene-4,4-dione, Sigma-Aldrich) dissolved in olive oil before the sham surgery (n = 6); the IR group received olive oil before the induction of renal IR (n = 16); the IR + ATX group received ATX before the induction of renal IR (n = 16). Mice in IR groups were sacrificed 12 h or 24 h post IR while mice in sham groups were sacrificed 24 h post sham surgery. Blood samples were collected for measurement of renal function while kidneys were collected for molecular analysis.

### Mice model of renal IR injury

Mice were anesthetized intraperitoneally with a mixture of ketamine (100 mg/kg) and midazolam (5 mg/kg). After anesthetization, mice were subjected to renal IR injury as previously described [21]. Briefly, after abdominal laparotomy, right kidney were exposed and removed. Left renal pedicle was exposed and clamped with vascular clamp for 45 min to induce ischemia. After clamp, vascular clamp was removed to induce reperfusion and the incision was closed in 2 layers. Sham-operated control animals underwent right kidney removal without occlusion of left renal artery.

### Measurement of renal function

Serum were separated by centrifuging blood samples and stored at −80°C until analysis of blood serum urea nitrogen (BUN) and urine creatinine (Cr). The concentrations of BUN and Cr were assessed in duplicated with a commercially available assay kit (BioAssay System, Hayward, CA, USA) according to the instructions.

### Histological analysis

Middle part of kidney were fixed in 4% formaldehyde, dehydrated, and paraffin embedded. Tissue sections (5 μm) were stained with hematoxylin and eosin (HE). Kidney sections were examined in a blinded manner and scored to evaluate the degree of injury. The score reflected the grading of tubular necrosis, cast formation, tubular dilation, and loss of brush border in 10 randomly selected, non-overlapping fields (200X) as following: 0, none; 1, ≤10%; 2, 11 to 25%; 3, 26 to 45%; 4, 46 to 75%; and 5, ≥76% [22,23].

### Immunofluorescent analysis

After dewaxing and rehydration, the tissue sections were incubated with 3% BSA/0.3% Triton X-100 for 30 min at room temperature. After draining this solution, the sections were incubated at room temperature with rabbit anti-myeloperoxidase (MPO, 1:100, Santa Cruz Biotechnologies, Santa Cruz, CA, USA), mouse anti-α smooth muscle actin (α-SMA) (1:100, Boster, Wuhan, China) overnight. Control tissue sections were similarly prepared except no primary antibody was added. After rinse with PBS, the sections were incubated with Alexa-594-conjugated secondary antibody (Life Science). After rinses with PBS, the slides were incubated with 4′,6-diamidino-2-phenylindole (DAPI, Life Science). The fluorescent was detected by confocal in darkness.

### Detection of apoptosis

Quantitative determination of apoptosis in kidney sections was assessed by a terminal transferase-mediated dUTP nick-end labeling (TUNEL) assay using an In Situ Cell Death Detection Kit (Roche, Basel, Switzerland). After dewaxing and rehydration, the tissue sections were permeabilized with 0.1% Triton X-100 for 10 min. Incubation with label solution was used to detect the apoptotic cells according to the instructions. Apoptotic score was achieved by counting the number of positive nuclei in 10 random fields.

### Measurement of superoxide dismutase (SOD) and malondialdehyde (MDA)

Renal SOD and MDA levels were determined under the guidance of the kit instructions (Jiancheng Bioengineering Institute, Nanjing, China). Briefly, renal tissues were homogenized. After centrifugation, SOD was determined using the xanthine oxidase method while MDA was determined by the thiobarbituric acid (TBA) reaction. Each assay was repeated three times. Protein concentration was determined by the BCA Protein assay (Pierce, Rockford, IL) using bovine serum albumin (BSA) as a standard.

### Determination of myeloperoxidase (MPO) activity

Commercial available kit (Jiancheng Bioengineering Institute) was used to detect MPO, an indicator of neutrophil infiltration, in the renal tissues according to the instruction. MPO activity was defined as the quantity of enzyme degrading 1 μmol of peroxide per minute at 37°C and was expressed in unit per milligram protein.

### Enzyme-linked immunosorbent assay *(ELISA)*

The supernatant from kidney homogenate was prepared for detecting the levels of TNF-α, IL-1β, and IL-6 with a commercial available ELISA kit following the instructions of the manufacturer (Uscn Life Science Inc, Wuhan, China). The absorbance was read on a microplate reader and the concentrations were calculated according to the standard curve. Protein content in the sample was determined by Coomassie blue assay and the results were corrected per microgram of protein.

### Statistical analysis

Data were analyzed using Prism 4 (GraphPad Software, San Diego, CA, USA) and expressed as mean ± standard deviation. Multiple groups were compared using one-way analysis of variance followed by the Tukey-Kramer test for post-hoc comparisons. Statistical significance was set at *P* < 0.05.

## Results

### ATX protects HETCs against H_2_O_2_-induced death

**Figure 1:**
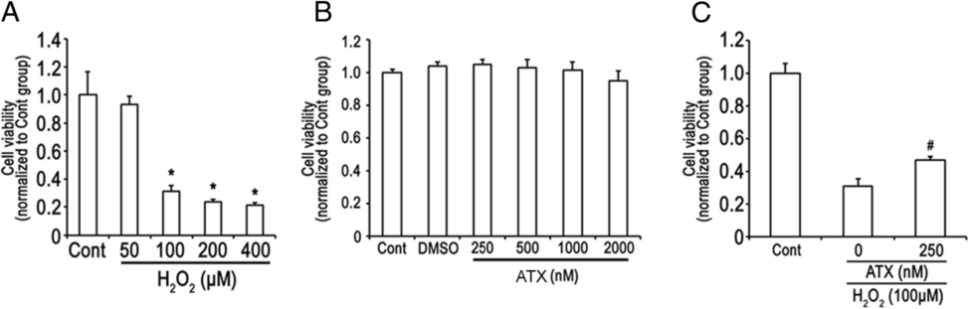
**Protective effect of ATX on H**_**2**_ **O**_**2**_ **-induced cell death. (A)** HTECs were incubated with different concentrations of H_2_O_2_ for 2 h. **(B)** HTECs were incubated with different concentrations of ATX for 24 h. **(C)** HTECs were pretreated with ATX (250 nM) for 24 h and then exposed to H_2_O_2_ (100 μM) for 2 h. *, P < 0.01 compared with control group, #, P < 0.01 compared with H_2_O_2_ group.

As shown in Figure 1, H_2_O_2_ decreased the cell viability in a dose dependent manner. The concentration of 100 μM was further used for the determination of H_2_O_2_-inudced cell damage in the present experiments. ATX at different concentration (250, 500, 1000, 2000 nM) did not show toxic effect on the cells (Figure 1B). The concentration of 250 nM was used for the further experiment. As shown in Figure 1C, ATX significantly increased the viability of HTECs exposed to H_2_O_2_.

### ATX preserves renal function and renal histology

**Figure 2:**
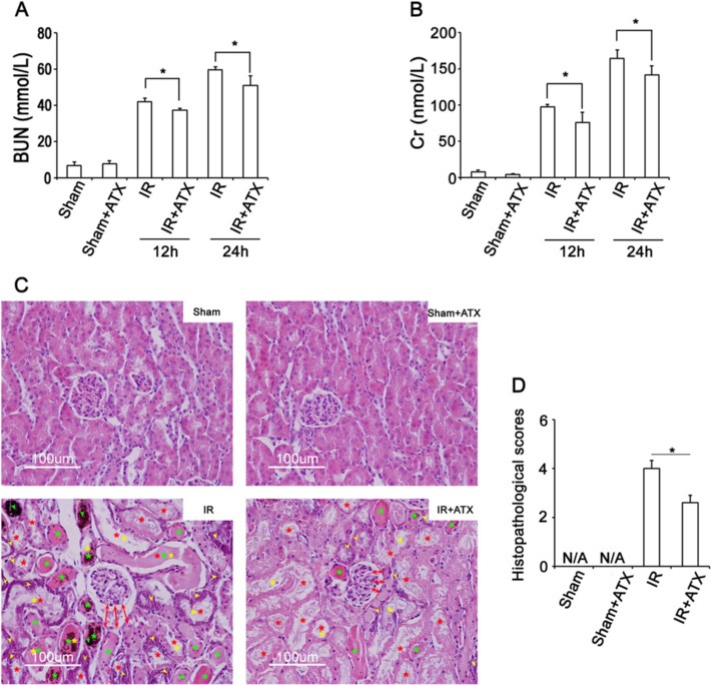
**ATX preserves renal function and histology after IR.** ***(*** **A-B)** Circulating levels of urea nitrogen (BUN) and creatinine (Cr) in different experimental groups (n = 6 in each group). **(C)** Representative images of HE staining of kidney sections in each experimental groups, showing significantly higher degree of tubular damage including tubular necrosis (yellow arrow), cast formation (green asterisk), dilatation of tubules (red asterisk), loss of brush border (yellow asterisk) in IR group. Please note that dilatation of Bowman’s capsule (red arrow), and accumulation of red blood cells in tubules (green arrow) could also be observed in IR groups, suggesting the damage to glomerular. **(D)** Results of total histopathological scores reflecting tubular damage in each group. *, P < 0.05 compared with IR group.

Two time points (12 h after and 24 h after IR procedure) were chosen to evaluate serial changes in serum levels of BUN and Cr. As shown in Figure 2A-B, both BUN and Cr levels were significantly higher in IR group than sham group and sham + ATX group. Pretreatment with ATX showed positive effect in preserving renal function, reflected by significantly reduced BUN and Cr levels in IR + ATX group. There was no significance between sham group and sham + ATX group.

To determine the effect of ATX on IR-induced renal injury, a histological scoring system base on the typical microscopic features of acute tubular damage including tubular necrosis and dilatation, cast formation, and loss of brush border was adopted (Additional file 1: Figure S2). At 24 hours after the IR procedure, the injury score was highest in IR group, significantly higher than sham group. The injury score in IR + ATX group was significantly reduced compared with IR group, suggesting the protective effect of ATX for IR-induced renal injury (Figure 2C-D).

### ATX alleviates oxidative stress

**Figure 3:**
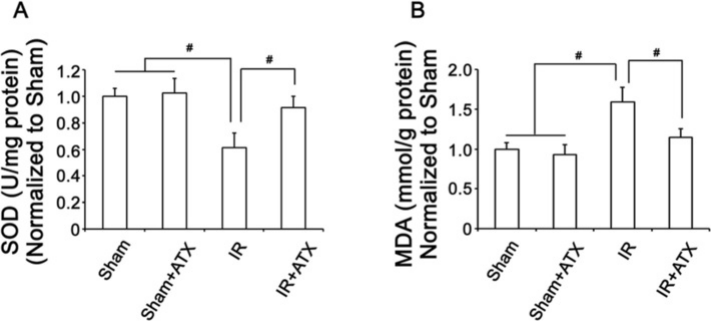
**ATX alleviates oxidative stress after IR.** Levels of SOD **(A)** and MDA **(B)** in the renal tissue in each experimental group. *, P < 0.05 compared with IR group.

Levels of SOD and MDA were used to evaluate oxidative stress in kidney. As shown in Figure 3, level of SOD was significantly higher while level of MDA was significantly lower in IR group compared with sham group, indicating oxidative stress in the kidney 24 h after IR procedure. Pretreatment of ATX reversed oxidative stress in IR + ATX group, reflected by significantly increased SOD level and decreased level of MDA compared with IR group.

### Effects ATX on reducing apoptosis in kidney after IR procedure

**Figure 4:**
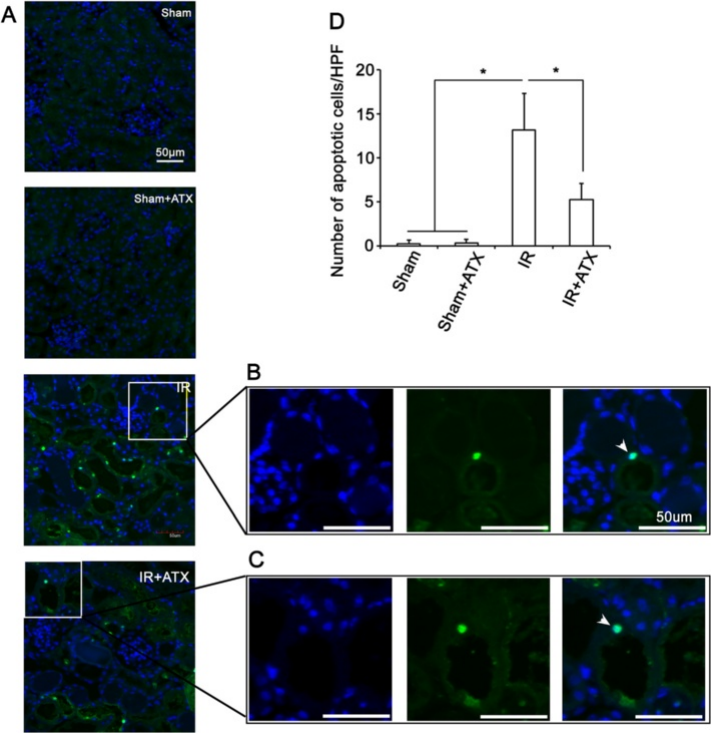
**ATX reduces tubular apoptosis after IR. (A)** Representative images of TUNEL staining of kidney sections in each experimental groups. **(B-C)** High magnification of the boxed area. White arrows show TUNEL positive cells. **(D)** Results of the number of apoptotic cells in kidney sections in each group. *, P < 0.05 compared with IR group.

To investigate IR associated apoptotic cells, we measured TUNEL positive cells in kidney tissues. As shown in Figure 4, 24 hours after IR, no apoptotic cells were observed in the kidney from sham group and sham + ATX group. The number of apoptotic cells increased significantly in IR group compared with sham group. In contrast, tissues from IR + ATX group contain a significantly smaller number of TUNEL-positive apoptotic cells.

### Effects of ATX on decreasing inflammation in kidney after IR

**Figure 5:**
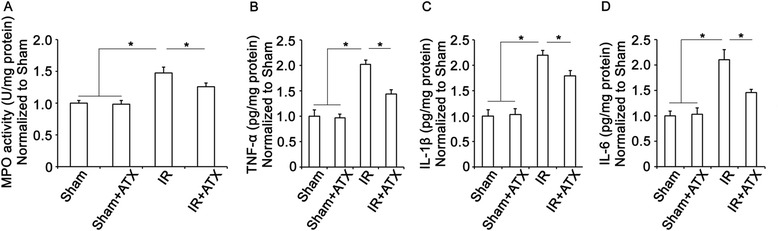
**Effects of ATX on decreasing inflammation in kidney after IR. (A)** Results of MPO activity in renal tissue in each experimental group. **(B-D)** Levels of TNF-α, IL-1β, and IL6 in renal tissues in each group. *, P < 0.05 compared with IR group.

MPO activity in kidney tissue and the protein levels of inflammatory cytokines including TNF-α, IL-1β, and IL-6 were used to evaluate inflammation in kidneys after IR procedure. As shown in Figure 5A, the MPO activity in IR group was significantly higher than that in sham group 24 h after IR, indicating neutrophil infiltration after IR procedure. Compared with IR group, ATX pretreatment produced a significant decrease of MPO activity.

ELISA detection showed significantly increased expressions of TNF-α, IL-1β, and IL-6 in IR group compared with that in sham group, suggesting inflammation in kidney 24 h after IR procedure. In the IR + ATX group, the levels of inflammatory cytokines decreased significantly compared with the IR group (Figure 5B).

### ATX reduces the expression of α-SMA in tubular cells after IR

**Figure 6:**
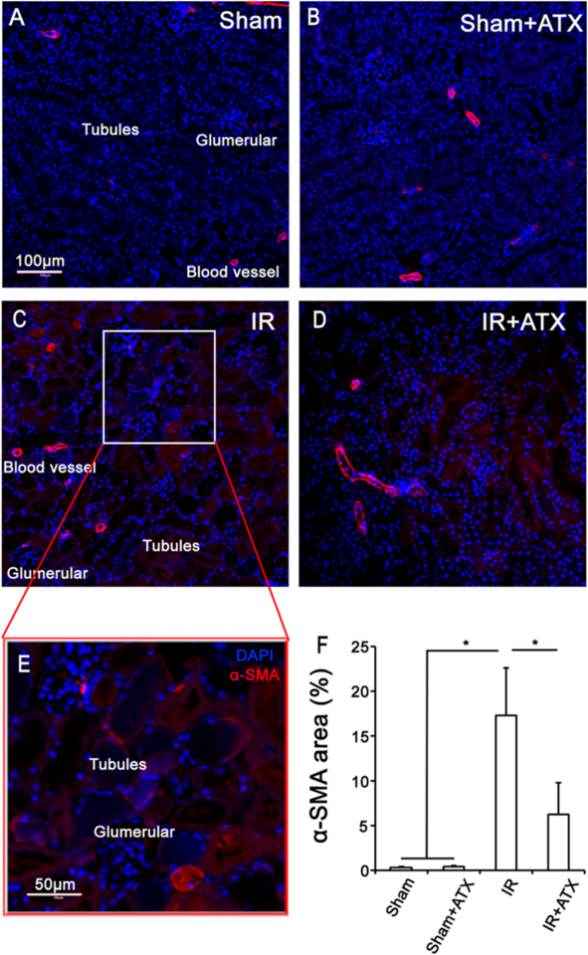
**Effects of ATX on reduces the expression of α-SMA in tubular cells after IR** ***.*** **(A-D)** Representative images of immunofluorescent staining of α-SMA of kidney sections in each group. **(E)** High magnification of the boxed area in C further shows the α-SMA expression in renal tissues. **(F)** Results of the expression of α-SMA in renal sections in each group. *P < 0.05 compared with IR group.

α-SMA positive myofibroblasts are the main actors in the pathogenesis of renal fibrosis. Because α-SMA production is modulated by ROS in some cell lines, we investigated the expression of this myofibroblast marker in our mice model of IR injury. As shown in Figure 6, almost none α-SMA expression could be detected within the interstitial space in the sham and sham + ATX group, while a significant increase in the expression of α-SMA was observed within the tubular areas of renal tissues 24 h post IR procedure. In contrast, the expression of this marker was significantly decreased in the IR + ATX group.

## Discussion

This study examined the effect of ATX pretreatment on IR-induced renal injury. Tissue distribution of ATX was studied previously in rodent after multiple-dosage regimens. The results revealed high accumulation of ATX in kidneys [24]. In the present study, ATX was dissolved in olive oil and administrated at a dosage of 5 mg/kg/day via oral gavage for 14 consecutive days before the induction of renal IR. The dosage of 5 mg/kg/day was selected based on our preliminary results that dosage of 10 mg/kg/day or 20 mg/kg/day did not show significantly enhanced efficacy in ameliorating renal function (Additional file 1: Figure S2).

ATX pretreatment leaded to an impressive protection in IR induced renal injury reflected by significantly increased viability of HTECs (Figure 1) and lower total histopathological score (Figure 2). As stated previously, the total histopathological score was obtained by summation of all scores given to tubular necrosis, cast formation, tubular dilation, and loss of brush border [22]. The histopathological evaluation revealed that total injury score was significantly ameliorated in ATX pretreated kidneys underwent IR as compared to non pretreated control. BUN and Cr, which are clinically used as markers to reflect renal function, were used in the present study to reflect renal function post IR. Although the functional differences between ATX pretreated group and non-treated control is significant (Figure 1), the decline of BUN or Cr in ATX pretreated groups was not consistent with that of histopathological score in ATX pretreated group. This might be because BUN and Cr are mainly used to reflect the filtration function of glomerular. Therefore, it gives us inspiration that more sensitive markers might be needed to fully reflect the renal injury after IR.

As well demonstrated, the similar molecular structure to β-carotenoid gives ATX the capacity to scavenge free radicals and protect oxidative damage to cell membranes, cells, and tissues [15]. ASX can stabilize free radicals by adding them to its long double-bond chain rather than donating an atom or electron to the radical [25]. In the present study, SOD and MDA, which are commonly used as biomarkers to reflect oxidative stress, were used to evaluate oxidative stress in kidney post IR. Significantly reduced levels of SOD and MDA in the ATX pretreated kidney compared with non-treated controls indicated the powerful free radical scavenging activity of ATX after IR injury (Figure 2), which was consistent with previous studies [17,18,20]. Combined with the preserved renal function and renal microstrucutre, it’s reasonable to speculate that ATX, a powerful antioxidant, might preserve renal function through reducing oxidative stress after IR. More importantly, the specific structure of ATX provides greater antioxidant properties than other antioxidants [26]. ATX contains two additional oxygenated groups on each ring structure, makes ATX highly polar. The polar end groups overlap the polar edges of the all membrane, while the non-polar middle fits the membrane’s non-polar interior. This allows ATX to neutralize free radical or other oxidant activity in both the non-polar center of the membrane, as well as along the polar boundary zones of the membrane (Additional file 1: Figure S1) [26,27]. Furthermore, since ATX stabilizes free radicals by adding them to its long double-bond chain rather than donating an atom or electron to the radical, it can resist chain reactions that occur when a fatty acid is oxidized, thus allowing it to scavenge or quench longer than antioxidants that cannot stop the chain reaction [25].

As stated in introduction, oxidative stress, a major contributor to the pathogenesis of IR, leads to tubular apoptosis/necrosis and triggers local inflammatory response in IR injury [7]. In the present study, tubular apoptosis, necrosis (Figure 1 and Figure 3) and inflammation (Figure 4) were shown to be prominent features 24 h post IR. Pretreatment of ATX significantly reduced tubular apoptosis/necrosis and inflammation after IR injury. Considering the crucial role of oxidative stress in inducing pathological changes of IR and the antioxidant properties of ATX, ATX might alleviate tubular necrosis/apoptosis and inflammation via scavenging free radicals.

Oxidative stress and inflammation caused by IR could induce EMT [9,28]. Although the role of EMT in the pathogenesis of end-stage renal fibrosis is debated, α-SMA expression by tubular cells has been suggested to be an early marker of progressive graft damage in kidney transplantation [29]. α-SMA is a specific marker for myofibroblasts, the main cellular type involved in tissue fibrosis [30]. In the present study, significantly increased α-SMA expression was detected in the tubular cells 24 h after IR injury, which is consistent with previous study using a swine model [31]. The over-expression of α-SMA in tubular cells could partially reflect tubular injury by oxidative stress and inflammation. There is an increasing body of evidence suggesting that IR injury not only causes acute tubular injury but also might be responsible for progressive renal damage leading to worse long-term function [32]. In the present study, pretreatment of ATX, in addition to reducing tubular apoptosis and inflammation, had protective effects in decreasing the expression of α-SMA (Figure 5). It might mainly due to the free radical scavenging activity of ATX, and subsequent decreased inflammation. Although the protective effects of ATX on long-term renal fibrosis was not determined in the present study, ATX might have positive effect in protecting kidney from fibrosis via regulating EMT early after IR injury.

## Conclusion

In conclusion, our data showed that pretreatment of ATX was effective in protecting IR-induced acute injury including tubular apoptosis/necrosis, inflammation, and EMT via scavenging free radicals (Additional file 1: Figure S4). Since ATX has been approved by the US FDA in 1999 as a dietary supplement [13], it might be a safe and effective strategy for kidney transplantation in preventing acute injury caused by IR.

## Additional file

Additional file 1: **Supplementary materials.**
