# Supplementary material for: Protective effects of astaxanthin against ischemia/reperfusion induced renal injury in mice
Source: J Transl Med. 2015 Jan 27;13:28. doi: 10.1186/s12967-015-0388-1 (PMC4323259; doi:10.1186/s12967-015-0388-1)
Supplement: Supplementary file 1 — Supplementary materials. [file 12967_2015_388_MOESM1_ESM.docx]

Protective effect of astaxanthin against ischemia-reperfusion induced renal injury in mice

**Supplementary materials:**

**
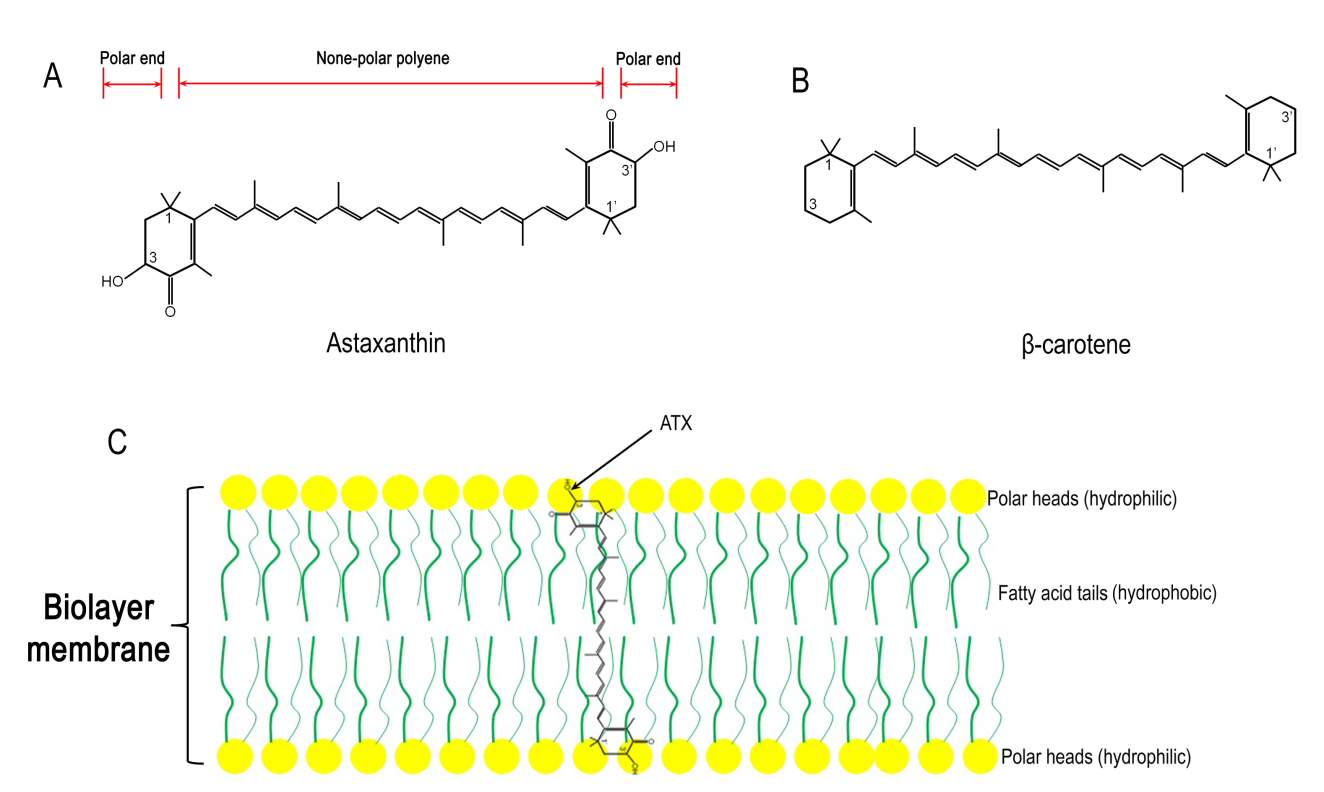
**

*Figure.S.1*: Molecular structure of astaxanthin (A) and β-carotene (B). C, Schematic of transmembranous alignment of ATX in biolayer cell membrane.


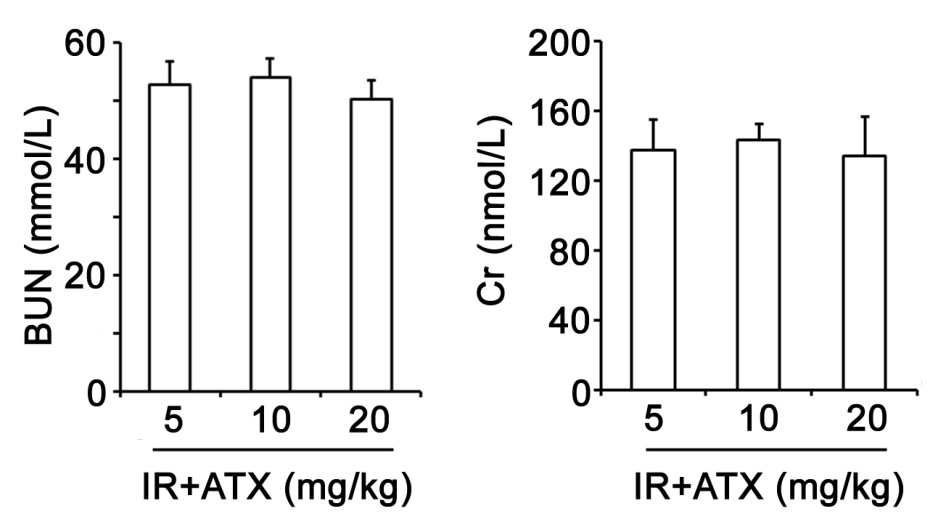


*Figure.S.2*: Levels of BUN and Cr in experimental groups treated with different dosage of ATX including 5mg/kg/day, 10mg/kg/day, and 20mg/kg/day.


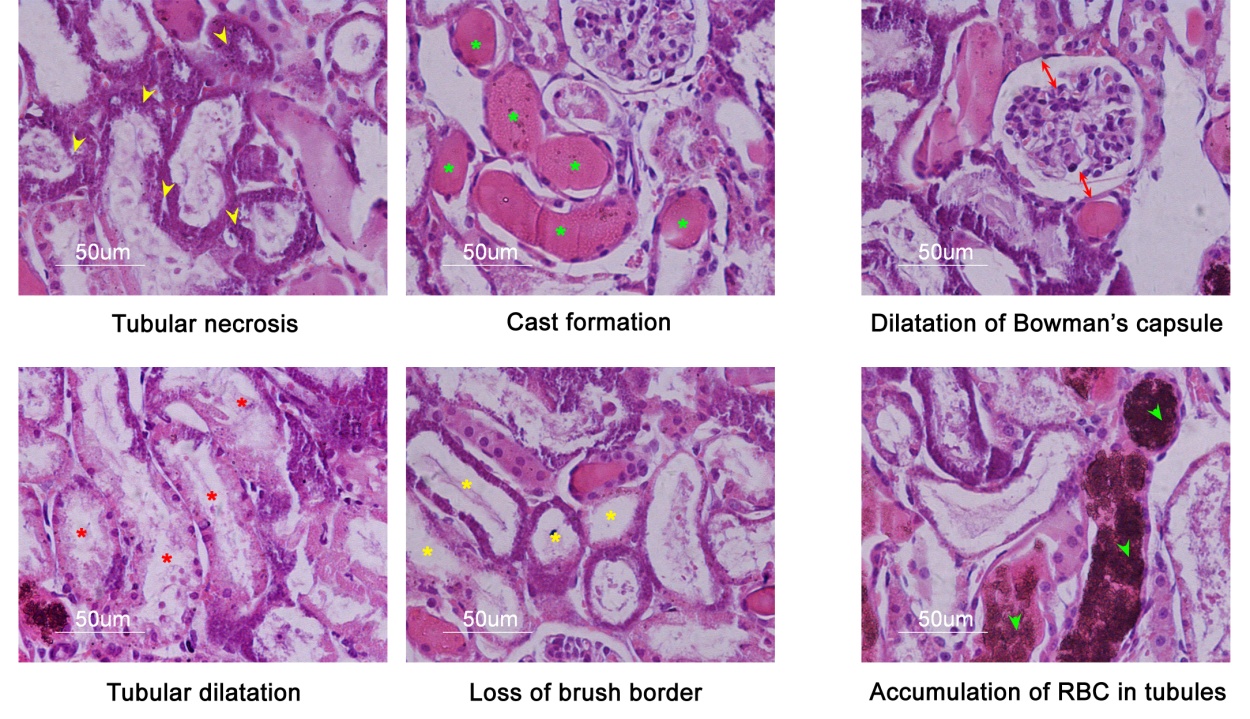


*Figure.S.3*: High magnification of kidney sections, showing the histopathological changes including tubular necrosis (yellow arrow), cast formation (green asterisk), dilatation of tubules (red asterisk), loss of brush border (yellow asterisk), dilatation of Bowan’s capsule (red arrow), and accumulation of red blood cells (RBC) in tubules (green arrow) in renal tissues 24h after IR.


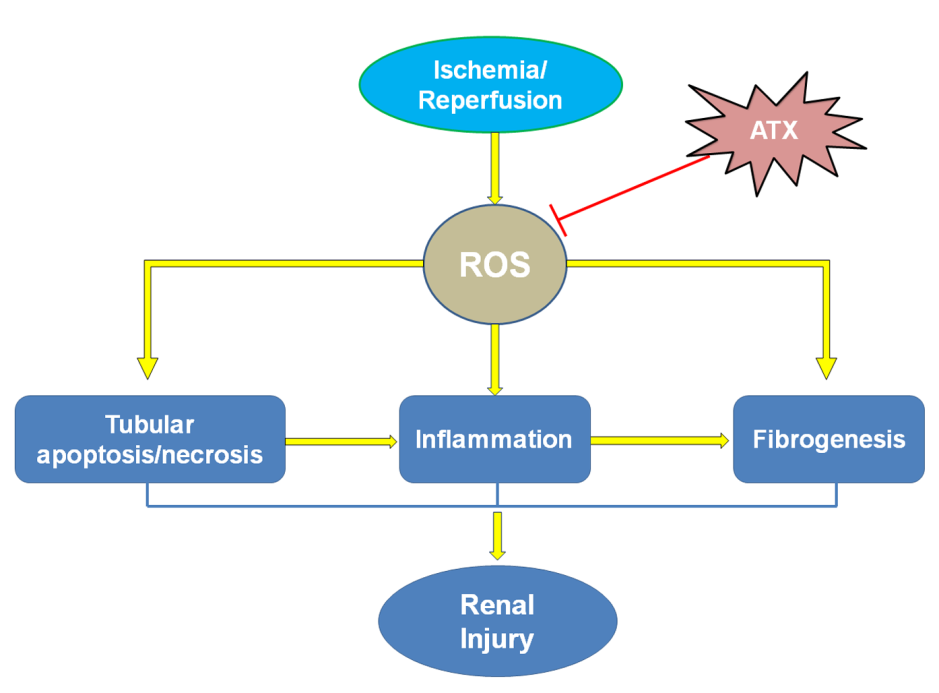


*Figure. S.4*: Schematic of the possible mechanisms involved in the protective effects of ATX for IR-induced renal injury.
